# Low-Temperature Plasma Activation of Biomaterials and Its Stability over Time and Post-Sterilisation Effects

**DOI:** 10.3390/ma19030643

**Published:** 2026-02-06

**Authors:** Piotr Trębacz, Mateusz Pawlik, Aleksandra Kurkowska, Karolina Wilk, Agata Piątek, Michał Czopowicz

**Affiliations:** 1 Department of Small Animal Surgery and Anesthesiology, Institute of Veterinary Medicine, Warsaw University of Life Sciences-SGGW, Nowoursynowska 159 C, 02-776 Warsaw, Poland; 2 Department of Biomaterials and Medical Devices Engineering, Faculty of Biomedical Engineering, Silesian University of Technology, 41-800 Zabrze, Poland; mateusz.pawlik@cabiomede.com (M.P.); aleksandra.kurkowska@cabiomede.com (A.K.); karolina.wilk@polsl.pl (K.W.); agata.piatek@polsl.pl (A.P.); 3 CABIOMEDE Ltd., 25-663 Kielce, Poland; 4 Division of Veterinary Epidemiology and Economics, Institute of Veterinary Medicine, Warsaw University of Life Sciences-SGGW, Nowoursynowska 159 C, 02-776 Warsaw, Poland; michal_czopowicz@sggw.edu.pl

**Keywords:** low-temperature plasma, surface wettability, steam sterilisation, plasma surface activation, implant surface modification, contact angle, biomedical implants

## Abstract

Low-temperature plasma (LTP) activation is increasingly used as a surface modification technique to enhance the wettability and biological performance of metallic implants. However, the stability of plasma-induced surface changes and their interaction with standard sterilisation procedures remain insufficiently understood. This study aimed to evaluate the effects of LTP activation, steam sterilisation, and their combination with the wettability of metallic implant materials, as well as the temporal stability of these effects. Samples manufactured from Ti6Al4V sheet, additively manufactured Ti6Al4V, and additively manufactured cobalt–chromium alloy were subjected to low-temperature plasma activation, steam sterilisation, or both procedures. Surface wettability was assessed by measuring the contact angle of canine blood droplets immediately after treatment and over a five-day observation period. Low-temperature plasma activation resulted in a substantial reduction in the contact angle for all tested materials, indicating a pronounced increase in surface wettability. However, this effect gradually diminished over time. Steam sterilisation alone moderately improved wettability and showed relatively stable effects. When steam sterilisation was applied after plasma activation, the plasma-induced enhancement was significantly attenuated and rapidly lost during storage. These findings demonstrate that while LTP activation effectively improves surface wettability, its benefits are highly time-dependent and strongly influenced by subsequent sterilisation. Plasma activation should therefore be performed immediately before implantation or combined with sterilisation and storage strategies that preserve surface modifications.

## 1. Introduction

An implant is defined as an object permanently placed in a patient during a surgical procedure and is not a suture, vascular clip, or staple [1]. Biomedical engineering is continuously advancing, particularly in the field of implantable biomaterials used in human and veterinary medicine. Implants, such as bone screws and plates, dental prostheses, and joint replacements, play a crucial role in treating injuries, degenerative conditions, and congenital disabilities. Despite their structural and mechanical advantages, these implants face significant challenges, including bacterial colonisation, inadequate osseointegration, and changes to surface properties induced by sterilisation methods [2].

Various surface modification methods have been developed to enhance surface energy and improve implant performance, such as chemical and electrochemical surface treatments, hydrophilic coatings, mechanical surface modifications, and low-temperature plasma (LTP) activation [3,4,5]. These techniques, either individually or in combination, play a crucial role in optimising the performance of orthopaedic implants by enhancing their interaction with live tissues and minimising the risk of implant failure [6,7]. Plasma is considered the fourth state of matter. Fluorescent lighting is among the earliest known commercial uses of plasma technology [8]. Plasma is a reactive gas mixture composed of ions, electrons, radicals, and neutral particles. Two types of plasma can be generated artificially by altering the physical properties of a neutral gas: thermal plasma, also known as hot/high-temperature/equilibrium plasma, and non-thermal plasma, also known as cold-atmospheric/low-temperature/non-equilibrium plasma [9]. The activation of biomaterial surfaces using plasma can be categorised into several distinct approaches, each with unique characteristics and applications. LTP activation operates at temperatures below 40 °C, enabling surface modifications without inducing thermal degradation [9,10,11,12,13,14,15]. The ability to modify material surfaces without affecting their bulk properties makes it particularly suitable for biomedical applications [16]. LTP activation technique modifies surface properties by increasing surface energy, improving wettability, and introducing functional oxygen-containing groups that enhance biocompatibility [16,17]. Additionally, plasma-induced chemical reactions facilitate surface functionalization, introducing hydrophilic groups such as hydroxyl (-OH), carboxyl (-COOH), and amine (-NH_2_) onto the biomaterial surface.

The wettability of an implant surface is a crucial factor that influences its interaction with the surrounding biological environment. Surface wettability is the ability of a liquid to maintain contact with a solid surface, resulting from the balance between adhesive forces at the liquid–solid interface and cohesive forces within the liquid. In practice, wettability is quantified by measuring the contact angle formed between the liquid and the surface. Lower contact angles indicate higher wettability (hydrophilicity), whereas higher angles indicate lower wettability (hydrophobicity). This method is widely used to evaluate the effectiveness of implant surface modifications and their potential interactions with surrounding biological fluids (Young’s equilibrium contact angle) [18]. In theory, the equilibrium contact angle is described by Young’s equation, which relates the interfacial free energies at the liquid–gas, solid–gas, and liquid–solid interfaces [19].

While a material’s mechanical strength is determined by its bulk properties, its surface characteristics dictate its interaction with living tissue [20]. Additionally, a hydrophilic surface reduces bacterial adhesion, thereby lowering the risk of biofilm formation and post-operative infections [21]. These effects are particularly critical in orthopaedic applications, where infection-related implant failure remains a significant concern. The biological relevance of implant surface wettability is further emphasised by evidence showing that hydrophilic surfaces promote more favourable early bone healing than hydrophobic ones. Implants with increased surface energy are consistently associated with enhanced protein adsorption, accelerated cell attachment, and greater early bone-to-implant contact, regardless of the grafting material used to fill the defect. These improvements in the initial healing phase translate into more stable early integration and may contribute to reduced healing times and improved long-term osseointegration. Collectively, these findings highlight the importance of engineering implant surfaces to achieve higher wettability, thereby optimising biological performance and clinical outcomes [22].

A critical concern in surgery is ensuring the sterility of implants before clinical use. Surgical site infections (SSIs) remain a significant challenge for surgical teams, accounting for up to a quarter of all healthcare-associated infections in humans [23]. In veterinary medicine, SSIs cause emotional and financial distress for owners and pose a serious threat to animal welfare. They are often perceived as an indication of the hospital’s or surgeon’s competence, adding to their reputational and psychological impact [1]. Steam sterilisation (autoclaving) is one of the most used methods and is effective at eliminating microorganisms by using pressurised steam. However, repeated steam sterilisation can have adverse effects, potentially altering the properties of implants and surgical instruments [24,25,26,27,28]. This phenomenon has already been observed in ≤10 autoclaving cycles [24,25,26,27]. The interaction between the LTP-activated surface and the steam sterilisation process is still unclear, as both processes can alter the surface’s chemistry, topography, and physicochemical properties [29]. Exposure to heat and moisture during autoclaving can degrade the hydrophilic functional groups introduced by plasma treatment, potentially reversing improvements in wettability [30,31,32,33].

The interaction between LTP-modified surfaces and steam sterilisation is a subject of ongoing research, as optimising sterilisation conditions is crucial for preserving plasma-induced enhancements while ensuring implant sterility [34,35]. This study aimed to evaluate the influence of LTP activation, steam sterilisation, and the combined effect of both procedures on the wettability of surfaces made from various materials, the stability of LTP activation over time, and its interaction with steam sterilisation.

## 2. Materials and Methods

### 2.1. Material and Sample Preparation

Three types of metallic samples were prepared for the study, representing materials and manufacturing routes commonly used in the production of orthopaedic implants.

- The first group consisted of Ti6Al4V sheet material with a thickness of 2 mm, rectangular specimens measuring 40 × 20 mm were fabricated from the sheet using abrasive waterjet cutting to minimise thermal influence on the material surface.
- The second group comprised circular Ti6Al4V specimens with a diameter of 15 mm, manufactured using selective laser melting (SLM) technology from metallic powder. The printing process used a layer thickness of 0.04 mm. After fabrication, the samples underwent heat treatment in accordance with the powder manufacturer’s recommended post-processing protocol.
- The third group consisted of circular CoCr alloy specimens with a diameter of 15 mm, also produced using SLM technology from metallic powder with a layer thickness of 0.04 mm. These samples were subjected to post-print heat treatment in accordance with the manufacturer’s specifications for the applied powder.

All specimens were sandblasted with 100–200 µm glass beads. This step reflects standard industrial finishing procedures routinely applied to orthopaedic implants manufactured by additive manufacturing or waterjet cutting. The objective of sandblasting was not to eliminate inherent topographical differences between rolled sheet materials and additively manufactured substrates, but rather to standardise surface finishing within each manufacturing route, thereby producing surfaces representative of real clinical end products before plasma activation and sterilisation.

### 2.2. Plasma Activation Process

**Figure 1:**
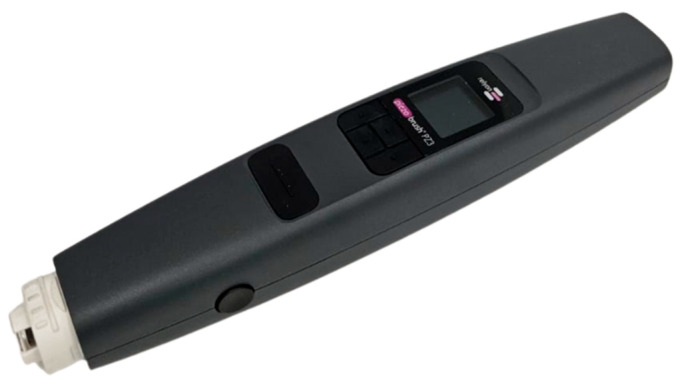
Piezobrush PZ3—handheld low-temperature plasma device for surface activation.

Samples were treated using the Piezobrush PZ3 (Relyon plasma GmbH, Regensburg, Germany) (Figure 1). This handheld device employed piezoelectric direct discharge (PDD) technology to generate LTP at room temperature without requiring external gases [36]. Plasma activation was performed for five consecutive days, with one set of samples activated per day. The plasma was applied at a fixed distance of 5 mm and for a controlled duration to ensure uniform treatment across all samples, with 5 s of activation at maximum PZ3 power per 1 cm^2^ of sample surface.

### 2.3. Sterilisation Procedure

To examine the impact of steam sterilisation on plasma-activated surfaces, a subset of the samples underwent autoclave. Samples were packaged separately for each cycle. The sterilisation process was conducted in an autoclave (Sun 12-II, Ningbo Mingtai Medical Instrument Co., Ltd., Ningbo, China) at 134 °C and 210 kPa, consistent with standard clinical practices, for a defined duration. All samples subjected to steam sterilisation were placed in loose paper–plastic sterilisation pouches commonly used in clinical practice. The samples were positioned such that the analysed surface did not come into contact with the pouch material. For specimens undergoing low-temperature plasma activation, the pouch was opened immediately before plasma treatment and subsequently resealed for storage, minimising direct handling and environmental contamination. This approach was selected to reproduce clinically relevant sterilisation, handling, and storage conditions. Samples were divided into three categories:

- LTP-activated and stored samples to assess the degradation of LTP effects over time (LTPA),
- LTP-activated and then steam-sterilised samples to evaluate the influence of autoclaving (LTPA&S),
- non-activated but sterilised samples serving as controls (S).

Additionally, a baseline sample was neither activated nor sterilised (NN), providing a reference for comparative analysis.

The potential impact of sterilisation on plasma-induced modifications was assessed by monitoring changes in surface chemistry and hydrophilicity. The high temperatures and humidity of autoclaving were expected to degrade oxygen- and nitrogen-containing functional groups introduced by plasma activation, potentially reducing the benefits of surface modification.

### 2.4. Wettability Testing Using Canine Blood

**Figure 2:**
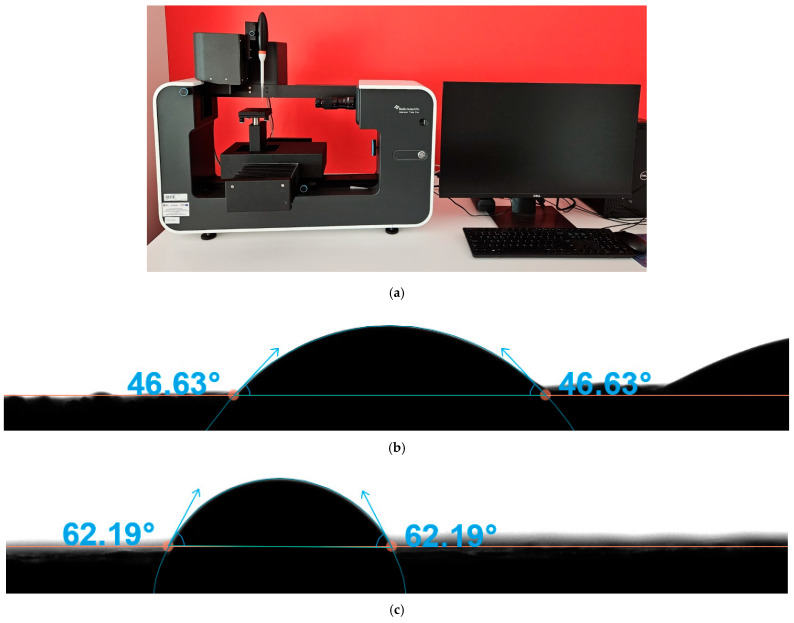
(**a**) A contact angle goniometer was employed to measure the wettability (θ) of Ti-sheet, Ti-3D, and CoCr-3D samples following activation and sterilisation treatments. (**b**) Blood contact angle (θ) measurement on the surface of the additively manufactured CoCr-3D sample, performed using the sessile drop method. (**c**) Blood contact angle (θ) measurement on the surface of the additively manufactured Ti-3D sample, performed using the sessile drop method.

Accordingly, contact angle measurements were conducted on blood samples obtained from adult dogs (both male and female) presenting for routine orthopaedic procedures (Figure 2a–c). Blood was collected during standard preoperative blood draws, and only samples from dogs without hematologic abnormalities were included to minimise biological variability. As stipulated by the Act of the Polish Parliament of 15 January 2015 on the Protection of Animals Used for Scientific or Educational Purposes (Journal of Laws 2015, item 266), ethics approval was not required for the use of residual clinical specimens [37]. The wettability of the treated surfaces was evaluated by measuring the contact angle (θ) of blood droplets, providing insights into the hydrophilic or hydrophobic nature of the implant surfaces. Surface wettability was assessed using the sessile drop method with an Attention Theta Flex optical tensiometer (Biolin Scientific, Espoo, Finland) and One Attention software (Nanoscience Instruments, Phoenix, AZ, USA, https://www.nanoscience.com/products/attension-tensiometers/oneattension-software/#overview, accessed on 13 August 2025). 1.5 mm^3^ blood droplets were used. Each measurement lasted 60 s, with a sampling frequency of 1 Hz. For each specimen, four independently deposited blood droplets were measured, each placed on a different region of the surface. These measurements represent technical replicates per sample and are intended to account for local surface variability.

A time-dependent analysis was performed to determine whether the effects of LTP activation gradually diminished over time and whether steam sterilisation altered the hydrophilic properties induced by plasma treatment. These findings are critical for optimising surface modification techniques to improve implant integration and performance in veterinary applications.

### 2.5. Statistical Analysis

Contact angle (θ) was summarised as the arithmetic mean and standard deviation (±SD). The association of materials and treatments with θ was analysed using the general linear model (GLM) with Tukey’s honest significant difference (HSD) test for differences between groups, i.e., combinations of 3 materials (Ti-sheet, Ti-3D, and CoCr-3D) and 4 treatments (NN, S, LTPA, LTPA&S). To retain acceptable power of statistical analyses, separate GLMs were developed to analyse: (I) differences in θ between groups on day 0 (the beginning of study GLM 1); (II) differences in θ between groups on day 5 (the end of study; GLM 2), (III) change of θ during the study period separately for each treatment (GLM 3—NN, GLM 4—S, GLM 5—LTPA, GLM 6—LTPA&S). For the mean differences between θ across groups on day 0 and day 5, Bonferroni-corrected 95% CIs were calculated from GLM 1 and GLM 2, respectively. To avoid Type I statistical error, pairwise comparisons were not performed on days other than day 0 and day 5. To quantify the rate of θ change during the study period, a linear regression equation was fit with an intercept coefficient (b0) estimating the initial θ and a slope coefficient (b1) estimating the rate of θ change per day. The coefficient of determination (R^2^) was used as a measure of goodness-of-fit. A significance level (α) was set at 0.05. Statistical analysis was performed in TIBCO Statistica 13.3 (TIBCO Software Inc., Palo Alto, CA, USA).

## 3. Results

The results of the contact angle (θ) measurements for the analysed materials and treatment variants are summarised in Table 1, presenting mean values with standard deviations obtained immediately after treatment and over the subsequent five days of observation.

### 3.1. Contact Angle of Three Non-Treated Materials (Group NN)

**Figure 3:**
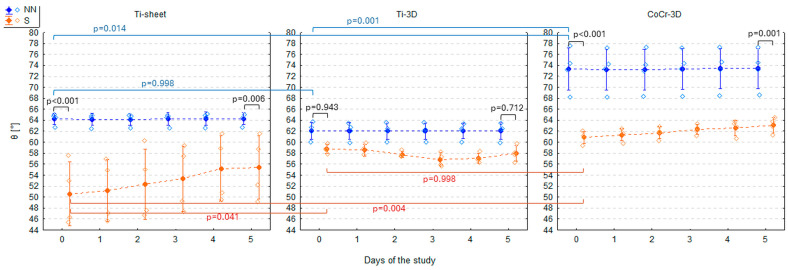
Contact angle (θ) of three tested materials (Ti-sheet, Ti-3D, and CoCr-3D) in the non-treated (NN) and steam-sterilised (S) groups in the study period presented as the arithmetic mean (dots), standard deviation (whiskers), and individual measurements (diamonds). *p*-values come from Tukey’s honestly significant difference (HSD) test.

On day 0, CoCr-3D had the highest θ (73.4 ± 3.9°), significantly higher than Ti-sheet (64.2 ± 1.0°; GLM 1 HSD test: *p* = 0.014) and Ti-3D (62.1 ± 1.6°; GLM 1 HSD test: *p* = 0.001). The θ of Ti-sheet and Ti-3D did not differ significantly on day 0 (HSD: GLM 1 HSD test: *p* = 0.998). The θ of all three materials in the NN group remained perfectly stable over 5 consecutive days (GLM 3: F5,54 = 0.01; *p* = 0.999) so that on day 5 the values of θ were virtually identical (Figure 3, Table 1).

### 3.2. Contact Angle of Three Materials After Steam Sterilisation (Group S)

Compared to the NN group, Ti-sheet and CoCr-3D from the S group had significantly lower θ on day 0 (GLM 1 HSD test: *p* < 0.001 for both)—it was lower by 13.7° (CI 95%: 5.2°, 22.1°) in Ti-sheet and by 12.5° (CI 95%: 4.1°, 20.9°) in CoCr-3D. In Ti-3D, θ was lower by 3.3°, but this difference was insignificant (GLM 1 HSD test: *p* = 0.943) (Figure 3, Table 1). As a result of the reduction of θ of CoCr-3D after steam sterilisation, θ became similar in Ti-3D and CoCr-3D (GLM 1 HSD: *p* = 0.998), while it was still significantly lower in Ti-sheet (vs. Ti-3D: GLM 1 HSD test: *p* = 0.041 and vs. CoCr-3D: GLM 1 HSD test: *p* = 0.004) (Figure 3, Table 1).

In the following days, θ in the group S did not change significantly (GLM 4: F5,54 = 0.61, *p* = 0.691). However, a very slight increase in θ could be observed in the Ti-sheet and CoCr-3D. As a result, on day 5, their θ values were still significantly lower than in the NN group but slightly less than on day 0—by 8.9° (CI 95%: 1.3°, 16.5°; GLM 2 HSD test: *p* = 0.006) in Ti-sheet and by 10.3° (CI 95%: 2.7°, 18.0°; GLM 2 HSD test: *p* = 0.001) in CoCr-3D. Despite some apparent fluctuations, no change was observed in Ti-3D—on day 5, θ was lower by 4.1° compared to the NN group (GLM 2 HSD test: *p* = 0.712) (Figure 3, Table 1).

Given an apparent (although statistically insignificant) increasing trend in Ti-sheet and CoCr-3D, a simple linear regression was fitted with days from sterilisation as the independent variable to estimate the rate of increase in θ for Ti-sheet and CoCr-3D. The slope coefficient proved to be insignificant in the Ti-sheet (*p* = 0.114) and significant in the CoCr-3D (*p* = 0.002), and the θ appeared to increase by approximately 0.5° per day (Table 2, Figure 3).

### 3.3. Contact Angle of Three Materials After LTP Activation

**Figure 4:**
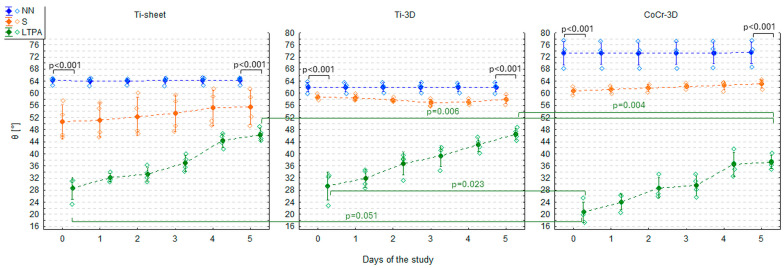
Contact angle (θ) of three tested materials (Ti-sheet, Ti-3D, and CoCr-3D) in the non-treated (NN), steam-sterilised (S), and low-temperature plasma activated (LTPA) groups in the study period presented as the arithmetic mean (dots), standard deviation (whiskers), and individual measurements (diamonds). *p*-values come from Tukey’s honestly significant difference (HSD) test.

Compared to the NN group, all three materials subjected to LTP activation had significantly lower θ on day 0 (GLM 1 HSD test: *p* < 0.001)—it was lower by 35.6° (CI 95%: 27.2°, 44.1°) in Ti-sheet, by 32.7° (CI 95%: 24.3°, 41.1°) in Ti-3D, and by as much as 52.7° (CI 95%: 44.2°, 61.1°) in CoCr-3D (Figure 4, Table 1). The most significant reduction in θ following LTP activation was observed in CoCr-3D. As a result, its θ became significantly lower than that of Ti-3D (GLM 1 HSD test: *p* = 0.023) and approached statistical significance when compared with Ti-sheet (GLM 1 HSD test: *p* = 0.051). However, given that non-treated CoCr-3D exhibited the highest baseline θ on day 0, its post-activation θ was only slightly lower than those of the two Ti-based materials, despite the most significant absolute decrease. The θ of Ti-sheet and Ti-3D did not differ significantly after LTP activation (GLM 1 HSD test: *p* = 0.999) (Figure 4, Table 1).

The effect of LTP activation proved to be unstable over time. A statistically significant increase in θ was observed during the days following LTP activation (GLM 5: F5,54 = 58.9; *p* < 0.001). The rate of this increase did not differ significantly between materials (GLM 5: F10,54 = 0.51; *p* = 0.879) and was pretty constant, at approximately 3–4° per day (Figure 4). Time elapsed since activation accounted for 80–85% of the variance in θ, indicating a perfect model fit (Table 3, Figure 4).

Despite significant constant increase of θ of all materials from the LTPA group in consecutive days, θ of all materials on day 5 was still significantly smaller than θ of materials from the NN group on day 5 (GLM 2 HSD test: *p* < 0.001)—it was lower by 18.0° (CI 95%: 10.3°, 25.6°) in Ti-sheet, by 15.5° (CI 95%: 7.9°, 23.1°) in Ti-3D, and by 36.1° (CI 95%: 28.5°, 43.8°) in CoCr-3D. Because on day 0, θ of CoCr-3D was smaller than θ of two Ti-materials and the increase rate of θ of LTP activated materials was pretty constant for all materials during the 5 days, on day 5 θ of CoCr-3D was still significantly smaller than θ of Ti-sheet (GLM 2 HSD test: *p* = 0.006) and Ti-3D (GLM 2 HSD test: *p* = 0.004) (Figure 4).

### 3.4. An Influence of Steam Sterilisation on the Contact Angle of Three LTP Activated Materials

**Figure 5:**
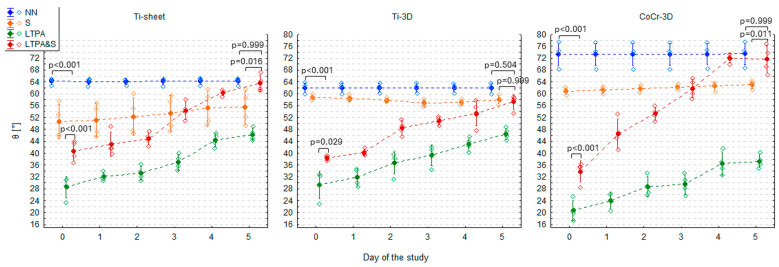
Contact angle (θ) of three tested materials (Ti-sheet, Ti-3D, and CoCr-3D) in the non-treated (NN), steam-sterilised (S), low-temperature plasma activated (LTPA), and low-temperature plasma activated and steam-sterilised (LTPA&S) groups in the study period presented as the arithmetic mean (dots), standard deviation (whiskers), and individual measurements (diamonds). *p*-values come from Tukey’s honestly significant difference (HSD) test.

Compared to the NN group, all three materials from the LTPA&S group had significantly lower θ on day 0 (GLM 1 HSD test: *p* < 0.001)—it was lower by 23.5° (CI 95%: 15.1°, 31.9°) in Ti-sheet, by 23.8° (CI 95%: 15.4°, 32.2°) in Ti-3D, and by 39.5° (CI 95%: 31.1°, 47.9°) in CoCr-3D. On the other hand, in all materials from the LTPA&S group, the θ was significantly higher than that from the LTPA group—it was higher by 12.2° (CI 95%: 3.7°, 20.6°; GLM 1 HSD test: *p* < 0.001) in Ti-sheet, by 8.9° (CI 95%: 0.4°, 17.3°; GLM 1 HSD test: *p* = 0.029) in Ti-3D, and by 13.1° (CI 95%: 4.7°, 21.6°; GLM 1 HSD test: *p* < 0.001) in CoCr-3D. It indicated that the reduction in θ achieved by LTP activation was substantially diminished by subsequent steam sterilisation. Nevertheless, in the LTPA&S group, θ was still significantly lower than in the S group—by 9.8° (CI 95%: 1.4°, 18.2°; GLM 1 HSD test: *p* = 0.006) in Ti-sheet, by 20.5° (CI 95%: 12.1°, 28.9°; GLM 1 HSD test: *p* < 0.001) in Ti-3D, and by 27.0° (CI 95%: 18.6°, 35.5°; GLM 1 HSD test: *p* < 0.001) in CoCr-3D (Figure 5, Table 1). This indicated that LTP activation performed before steam sterilisation achieved better wettability than steam sterilisation alone, especially in 3D-printed materials.

**Figure 6:**
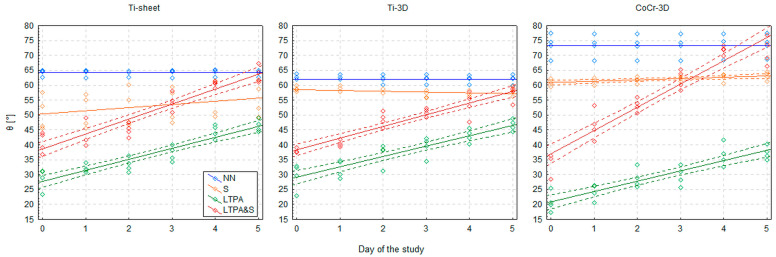
Linear regressions modelling the change of the contact angle (θϑ) of three tested materials (Ti-sheet, Ti-3D, and CoCr-3D) as a function of time in the steam-sterilised (S), low-temperature plasma activated (LTPA), and low-temperature plasma activated and steam-sterilised (LTPA&S) groups. If the slope coefficient was significant, 95% confidence intervals of the regression line were presented (broken lines).

As in the case of sole LTP activation, the effect of LTP activation followed by steam sterilisation proved unstable over time. A consistent, statistically significant increase in θ was observed (GLM 6: F5,54 = 146.8; *p* < 0.001), but the rate of this increase differed significantly between materials (GLM 6: F10,54 = 8.91; *p* < 0.001) (Figure 5). The steepest rise in θ, by approx. 8° a day occurred in CoCr-3D, while in both Ti-based materials, it was approximately. 4–5°. Time elapsed since activation and sterilisation accounted for 88–91% of the variance in θ, indicating a perfect model fit (Table 4, Figure 6).

As a result of higher baseline θ values on day 0 in the LTPA&S group and more rapid increase of θ in following days (as shown by slope coefficients), the θ values similar to the values from the NN group were reached on day 4 in the case of Ti-sheet and CoCr-3D, and on day 5 in the case of Ti-3D (Figure 5 and Figure 6, Table 1). Finally, on day 5, there was no significant difference in the θ values between the NN and the LTPA&S group (GLM 2 HSD test: Ti-sheet: *p* = 0.999; Ti-3D: *p* = 0.504; CoCr-3D: *p* = 0.999).

Moreover, rapidly increasing θ in the LTPA&S group reached values observed in the S group already on day 3 in the case of Ti-sheet and CoCr-3D, and finally on day 5 the θ values were significantly higher than in the S group—by 8.2° (CI 95%: 0.5°, 15.8°; GLM 2 HSD test: *p* = 0.016) in Ti-sheet and by 8.5° (CI 95%: 0.9°, 16.1°; GLM 2 HSD test: *p* = 0.011) in CoCr-3D. Only in Ti-3D θ values were similar between the LTPA&S and the S group (GLM 2 HSD test: *p* = 0.999) (Figure 5 and Figure 6, Table 1).

## 4. Discussion

LTP activation is an effective surface engineering technique for metallic implant materials, substantially increasing surface wettability. It can promote cell adhesion and tissue regeneration without leaving any residual surface energy or chemical impurities [38,39]. Furthermore, numerous studies have demonstrated that bacterial adhesion to plasma-treated titanium surfaces is significantly lower than to stainless steel or standard titanium materials [40,41]. Our study provides several interesting observations regarding the wettability of implant materials. First, the natural wettability of CoCr-3D material is worse (i.e., the θ is larger by approx. 10°) than the natural wettability of Ti-based materials (both steel sheet and 3D printed). Secondly, steam sterilisation slightly improves wettability (reduces θ by 10–15°), and this effect remains relatively stable for 5 days after sterilisation (as observed in Ti-sheet and CoCr-3D). Thirdly, LTP activation dramatically improves wettability (reduces θ by 35–50°). Similar results were reached by Harda et al. [42]. They also found that applying handheld low-temperature plasma enhanced the hydrophilicity of the Ti disks. In our study, this effect remained unstable for 5 days after LTP activation, and θ gradually increased at a rate of 3–4° per day. Finally, we discovered that steam sterilisation performed after LTP activation significantly attenuates the plasma-induced modification (reducing θ by 25–40°) and leads to rapid wettability loss over time. While an initial improvement in contact angle is still detectable, this effect is volatile and entirely disappears by the fifth day of storage. Notably, after this period, the wettability of activated and subsequently steam-sterilised materials may be inferior (i.e., characterised by a higher contact angle) to that achieved by steam sterilisation alone, as demonstrated for Ti-sheet and CoCr-3D. It should be noted that both surface chemistry and surface topography influence surface wettability, and residual differences in roughness between sheet-based and additively manufactured substrates may contribute to baseline differences in contact angle values. Additive manufacturing inherently introduces macro- and mesoscale surface features resulting from the layer-by-layer fusion process, which are not entirely removed by post-processing, such as sandblasting.

However, surface roughness represents a static characteristic under the applied experimental conditions and does not change during storage or following steam sterilisation. In contrast, the pronounced time-dependent increase in contact angle observed after low-temperature plasma activation, as well as the rapid attenuation of plasma-induced hydrophilicity following steam sterilisation, cannot be explained by roughness-related mechanisms alone (e.g., Wenzel-type effects). These findings strongly indicate that the observed wettability changes are governed predominantly by surface chemical and energetic modifications induced by plasma treatment and altered by sterilisation, rather than by differences in physical topography.

To assess the biological relevance of surface modifications, wettability tests were performed using fresh canine blood rather than deionised water or synthetic blood analogues. Given the lack of veterinary literature on implant wettability, using canine blood in our study could be valuable for small-animal surgery. While water contact angle measurements are commonly used to characterise surface energy, they do not fully replicate the complex interactions that occur at the implant–tissue interface in vivo [43,44,45]. In clinical settings, metallic implants are immediately exposed to blood and plasma proteins upon insertion, and the initial adsorption of proteins, platelets, and other blood components governs subsequent biological cascades, including coagulation, cellular recruitment, and early stages of osseointegration. Surface wettability toward blood has been shown to influence protein adsorption and platelet behaviour, which, in turn, are critical determinants of bone healing and implant integration in orthopaedic and dental applications [43,44,45]. Even relatively small differences in contact angle may be biologically relevant, as early blood-material interactions are susceptible to surface wettability and surface energy. Previous studies have shown that subtle changes in wettability can influence protein adsorption and conformation, thereby affecting early biological events at the implant interface [46]. These early processes play a key role in subsequent healing and osseointegration. As mentioned above, we used the blood as a testing medium in this experiment to provide a more physiologically relevant measure of surface–fluid interactions than water alone, demonstrating how surface treatments affect blood spread and thereby early events in the host response (e.g., protein adsorption, clot formation) that are not captured by water wettability tests alone.

This study used a small, handheld cold plasma generator. According to the manufacturer, this device was selected due to its precision, ease of use, and capacity for localised surface modification. Plasma was introduced to the implant surfaces to enhance their wettability and surface reactivity. These features could be helpful in clinical settings. Handheld cold plasma generators are now widely used in medicine for disinfecting water and surfaces, as well as for treating chronic wounds. They are replacing large laboratory generators, which are cumbersome and require additional devices (e.g., a vacuum pump) and a separate power supply [47]. In our study, materials subjected solely to LTP activation (without steam sterilisation) exhibited the best wettability. However, as sterilisation is an essential and inevitable element of pre-surgical procedures, this observation is rather theoretical. In practice, the best wettability is achieved when the material is activated and steam-sterilised immediately before use. Storage of activated material is associated with the gradual worsening of wettability. This process appears to be a gradual extrapolation of the regression line beyond the 5-day study period, suggesting that the contact angle would return to baseline values for the NN group after approximately 10 days. This estimate, however, is only an extrapolation, which is true only if a linear increasing trend holds and estimated slope coefficients are sufficiently accurate. Therefore, it should be treated with the highest caution. Our study showed that storing activated and steam-sterilised material is contraindicated, as wettability deteriorates rapidly, ultimately resulting in lower wettability than if the material had not been activated at all. Interestingly, steam sterilisation itself slightly improves wettability, at least for some materials, and this effect appears to be stable. In the CoCr-3D, if the further increasing trend beyond 5 observed days remains stable and the estimated slope coefficient is sufficiently accurate, θ is expected to reach values from the NN groups after approx. 4 weeks. This estimate, however, is only an extrapolation that should be treated with the utmost caution.

In this experiment, the observation period was relatively short, limiting the ability to capture both the hydrophobic recovery phenomenon and the logistical constraints associated with custom-made orthopaedic implants. This phenomenon involves a gradual decrease in the wettability of activated titanium and other materials over several days. This is attributed to various mechanisms, such as surface contamination, neutralisation of trapped charges, and desorption of low-molecular-weight fragments [48]. Conversely, custom-made implants are usually produced to order and delivered directly to the ordering surgeon shortly after final processing. In this context, prolonged storage over weeks or months is uncommon. A five-day timeframe is clinically realistic, encompassing final manufacturing, packaging, transportation and preparation for implantation following plasma activation. Adhering to this timeframe can help to avoid the influence of the hydrophobic recovery phenomenon on the results.

In addition to plasma activation, several other strategies for modifying implant surfaces have been developed to enhance wettability and promote interaction with living tissue, such as osseointegration. For example, sandblasting combined with acid etching creates micro- and nanoscale roughness on the surface of titanium and Ti-alloy implants, increasing surface energy and improving hydrophilicity and early cell attachment. Further chemical modifications, such as anodic oxidation and the application of hydroxyapatite or calcium phosphate coatings, further alter surface chemistry and topography, leading to increased wettability, enhanced protein adsorption, and improved cell adhesion [4,49]. In this study, we found that LTP was a helpful tool. However, the study also showed that the practical value of plasma activation depends heavily on subsequent processing steps. Hot steam sterilisation is an aggressive physicochemical process. Even stainless steel implants can be damaged by autoclaving [28]. However, steam sterilisation significantly attenuates plasma-induced surface modifications and accelerates the recovery of hydrophobicity. When plasma activation is followed by autoclaving and storage, the resulting surface properties rapidly converge toward those of non-activated materials, effectively negating the benefits of activation. In this context, plasma activation performed before steam sterilisation cannot be considered a robust or durable surface modification strategy for implants intended for delayed clinical use.

Despite the interesting results obtained, our experiment had some limitations. For example, we did not examine sterilisation and storage strategies that are compatible with plasma-activated surfaces. Steam sterilisation is the most widely available and popular method of sterilising metal objects in veterinary clinical practice. Further investigation is warranted into low-temperature sterilisation methods, such as ethylene oxide, gamma irradiation and hydrogen peroxide plasma, as well as wet storage approaches. Additionally, the relationship between wettability retention and biological outcomes, including protein adsorption, cellular response, antibacterial properties, and in vivo performance, requires further exploration.

This experiment also did not investigate the chemical composition, topography or roughness of the implant materials’ surfaces, nor the combined effects of plasma activation and different sterilisation methods on their long-term performance.

In addition, the surface activation effects of a handheld device were not compared with those of more efficient plasma vacuum systems. This experiment aimed to evaluate the feasibility of incorporating an easy-to-use, handheld, low-temperature plasma activation device into clinically realistic orthopaedic workflows, particularly for custom-made implants. Benchmarking different plasma technologies was not the focus.

## 5. Conclusions

LTP activation should be regarded as a time-sensitive and process-dependent surface treatment. Its beneficial effects can only be fully exploited if activation is performed immediately before implantation. The routine sequence of plasma activation followed by steam sterilisation and dry storage does not provide a meaningful long-term advantage and should be reconsidered in implant preparation protocols.

The process applies to both titanium- and cobalt–chromium-based substrates, including additively manufactured components, and effectively compensates for unfavourable baseline surface characteristics. From a materials engineering perspective, LTP-activation offers a rapid, low-energy, and non-destructive method for tailoring implant surface properties relevant to early biological interactions.

## Figures and Tables

**Table 1:** Arithmetic mean ± standard deviation (SD) of contact angle (θ) [°] of 3 materials subjected to 4 different treatments, measured just after the treatment and then for 5 consecutive days.

		Day of the Study					
Material	Treatment *	0	1	2	3	4	5
Ti-sheet	NN	64.24 ± 1.02	64.17 ± 1.12	64.14 ± 1.04	64.22 ± 1.12	64.29 ± 1.18	64.31 ± 1.13
Ti-sheet	S	50.58 ± 5.80	51.16 ± 5.62	52.36 ± 6.43	53.34 ± 5.97	55.19 ± 5.93	55.41 ± 5.73
Ti-sheet	LTPA	28.61 ± 3.61	32.08 ± 1.39	33.29 ± 2.33	36.93 ± 2.62	44.41 ± 2.33	46.33 ± 2.11
Ti-sheet	LTPA&S	40.77 ± 3.39	42.98 ± 4.07	44.89 ± 2.14	54.32 ± 2.94	60.40 ± 1.05	63.58 ± 2.78
Ti-3D	NN	62.09 ± 1.57	62.04 ± 1.54	62.04 ± 1.48	62.04 ± 1.46	62.04 ± 1.39	62.04 ± 1.56
Ti-3D	S	58.76 ± 0.82	58.56 ± 1.05	57.74 ± 0.61	56.81 ± 1.22	57.09 ± 0.89	57.96 ± 1.42
Ti-3D	LTPA	29.42 ± 4.62	31.85 ± 2.97	36.81 ± 3.85	39.31 ± 3.38	42.98 ± 2.30	46.56 ± 1.86
Ti-3D	LTPA&S	38.28 ± 0.81	40.26 ± 1.15	48.48 ± 2.39	50.89 ± 1.33	53.51 ± 4.38	57.30 ± 2.64
CoCr-3D	NN	73.35 ± 3.88	73.24 ± 3.73	73.20 ± 3.74	73.32 ± 3.71	73.42 ± 3.66	73.45 ± 3.64
CoCr-3D	S	60.88 ± 1.12	61.35 ± 1.13	61.75 ± 1.08	62.30 ± 0.88	62.60 ± 1.40	63.13 ± 1.38
CoCr-3D	LTPA	20.70 ± 3.38	24.14 ± 2.70	28.72 ± 3.31	29.56 ± 3.38	36.52 ± 3.86	37.33 ± 2.27
CoCr-3D	LTPA&S	33.84 ± 3.66	46.55 ± 4.97	53.34 ± 2.23	61.81 ± 3.16	71.82 ± 1.40	71.61 ± 4.66

* NN—non-treated, S—steam-sterilised, LTPA—low-temperature plasma activated, LTPA&S—low-temperature plasma activated and steam-sterilised.

**Table 2:** Coefficients of simple linear regression modelling the change of the contact angle (θ) as a function of time in the steam-sterilised (S) group.

	Intercept Coefficient	Slope Coefficient		
Material	b_0_ (CI 95%)	b_1_ (CI 95%)	*p*-Value	R^2^
Ti-sheet	50.3° (46.3°, 54.4°)	1.01° (−0.27°, 2.39°)	0.114	0.111
CoCr-3D	60.9° (60.1°, 61.7°)	0.44° (0.18°, 0.71°)	0.002	0.356

**Table 3:** Coefficients of simple linear regression modelling the change of the contact angle (θ) as a function of time in the low-temperature plasma activated (LTPA) group.

	Intercept Coefficient	Slope Coefficient		
Material	b_0_ (CI 95%)	b_1_ (CI 95%)	*p*-Value	R^2^
Ti-sheet	27.7° (25.7°, 29.7°)	3.7° (3.0°, 4.4°)	<0.001	0.858
Ti-3D	29.1° (26.9°, 31.4°)	3.5° (2.7°, 4.2°)	<0.001	0.808
CoCr-3D	20.8° (18.5°, 23.2°)	3.5° (2.7°, 4.2°)	<0.001	0.796

**Table 4:** Coefficients of simple linear regression modelling the change of the contact angle (ϑ) as a function of time in the low-temperature plasma-activated and steam-sterilised (LTPA&S) group.

	Intercept Coefficient	Slope Coefficient		
Material	b_0_ (CI 95%)	b_1_ (CI 95%)	*p*-Value	R^2^
Ti-sheet	38.6° (36.1°, 41.1°)	5.0° (4.2°, 5.8°)	<0.001	0.880
Ti-3D	38.3° (36.4°, 40.3°)	3.9° (3.3°, 4.6°)	<0.001	0.878
CoCr-3D	37.0° (33.7°, 40.3°)	7.8° (6.7°, 8.9°)	<0.001	0.910

## Data Availability

The original contributions presented in this study are included in the article. Further inquiries can be directed to the corresponding author.

## Abbreviations

The following abbreviations are used in this manuscript:

SSIs	Surgical Site Infection
LTP	Low-Temperature Plasma
LTPA	Low-Temperature Plasma Activated
LTPA&S	Low-Temperature Plasma Activated and Steam-Sterilized
PDD	Piezoelectric Direct Discharge
SLM	Selective Laser Melting
Ti6Al4V	Titanium–Aluminum–Vanadium alloy
CoCr	Cobalt–Chromium alloy
NN	Non-Treated
S	Steam-Sterilized
GLM	General Linear Model
HSD	Honest Significant Difference
CI	Confidence Interval
SD	Standard Deviation
